# Retrospective analysis of incidence, fracture pattern and treatment of zygomatic complex fracture in a dental teaching institute in India

**DOI:** 10.3389/froh.2025.1674963

**Published:** 2025-10-09

**Authors:** Somya Pande, Uday Londhe, Kalyani Bhate, Aditya Jape

**Affiliations:** Dr D.Y. Patil Dental College and Hospital, Dr D.Y. Patil Vidyapeeth, Pimpri, Pune, India

**Keywords:** zygomaticomaxillary complex fractures, ZMC, treatment strategies, surgical management, complications, paraesthesia

## Abstract

**Background:**

Zygomatic complex fractures (ZMC) are highly prevalent facial fractures. Several treatment strategies are available in the literature for managing ZMC fractures.

**Aim:**

To evaluate the incidence, fracture patterns, complications, and management strategies for ZMC fractures in one institution.

**Material and methods:**

The data regarding 100 ZMC patients were collected retrospectively, and details such as aetiology, site of the ZMC fracture, type of fracture, associated injuries, clinical findings, treatment with conservative or surgical intervention, type of incisions used, number of fixations used, and any complications encountered were reviewed and analyzed.

**Results:**

Violence was the predominant cause of ZMC fractures, affecting patients mainly in the 20–40 and over 60 age groups equally. For fractures without displacement, conservative management was effective. When surgery was required, fixation strategies varied using one-point, two-point, or three-point fixation depending on the severity and displacement of the fracture. Importantly, we observed that factors such as age, gender, or the cause of injury did not significantly affect the occurrence of paraesthesia before or after treatment.

**Conclusion:**

In conclusion, the choice of treatment modalities should be tailored to the specific fracture pattern and patient needs to ensure optimal outcomes.

## Introduction

Midface fractures represent a challenge for medical practitioners due to the severe esthetic and functional consequences. Zygomatic bone fractures are a pathology that challenges surgeons worldwide, representing the most frequent type of midface fracture in our geographical area (1, 2). They are the second most common type of mid-facial fracture, accounting for about a quarter of all facial bone injuries (3). The complexity of these fractures is directly proportional to the aetiology, direction and kinetic energy of the wounding agent, its acceleration speed change, and not least, to the surface of contact and the duration of the impact with the recipient. These fractures usually result from incidents such as accidents, falls, or physical assaults. The ZMC not only provides structural support but also plays a vital role in defining facial aesthetics by shaping the midface width and accentuating cheek prominence (4). Clinically, patients with ZMC fractures often present with symptoms such as double vision (diplopia), sunken eyeballs (enophthalmos), bruising under the eye (subconjunctival ecchymosis), flattening of the cheek, misalignment of the bite (gagging of occlusion), and sensory disturbances. Swelling in the midface region can also lead to noticeable cosmetic deformities (5). One common neurological symptom is paraesthesia in the area served by the infraorbital nerve.

Treatment options for ZMC fractures vary from conservative management to surgical procedures. Non-displaced fractures without functional issues are typically managed conservatively. Surgery is usually required when fractures are displaced, unstable, or fragmented into multiple pieces (6). Over 70% of ZMC fractures are treated with surgical methods like open reduction and internal fixation (7). Managing ZMC fractures poses a complex challenge because these injuries often involve both the maxilla and zygomatic bone, which are essential parts of the midface. The main goal is to restore proper three-dimensional alignment and stability, while also addressing any accompanying injuries to the infraorbital rim and orbital floor (8, 9). The literature describes various surgical techniques and fixation methods, chosen based on the extent and severity of the fracture, and whether the orbital floor is involved. Each approach has its benefits and limitations (8, 9). Typical fixation devices include external pins, lag screws, K-wires, transosseous wires, mini dynamic compression plates (DCP), miniplates, and microplates (10, 11). Despite the common occurrence of ZMC fractures, no standardised protocol exists among maxillofacial surgeons. Additionally, there is a lack of comprehensive research analysing the incidence, fracture patterns, and best management strategies for these injuries. This study aims to fill that gap by evaluating the incidence, fracture patterns, complications, and management approaches for ZMC fractures at one institution.

## Materials and methods

The present single-centre retrospective study was conducted in Dr. D.Y. Patil Dental College and Hospital, Pimpri, Pune, India. The data from 100 patients aged 20 years or older who were clinically and radiologically diagnosed with ZMC fractures and treated during the study period from July 2020 to July 2025 were retrospectively collected. Prior to Study commencement, approval was obtained from the Ethical Committee from Dr. D.Y. Patil Dental College and Hospital, Pimpri, Pune, India. (DYPDCH/DPU/EC/583/193/2023).

Parameters such as aetiology, site of the ZMC fracture, type of fracture, associated injuries, clinical findings, treatment with conservative or surgical intervention, type of incisions used, number of fixations used, and any complications encountered were reviewed and analysed.

## Results

**Figure 1:**
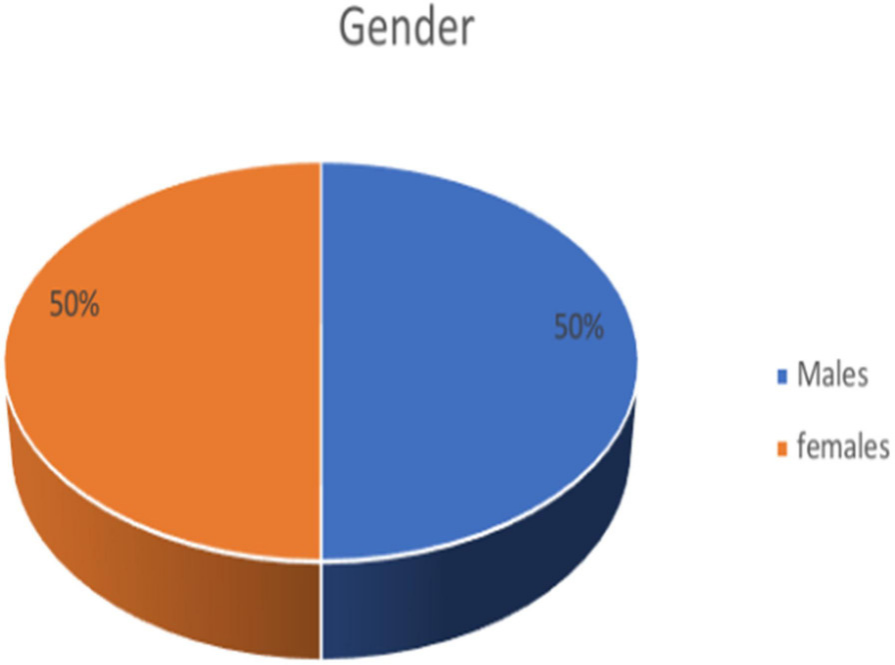
Distribution of the study participants based on gender.

**Figure 2:**
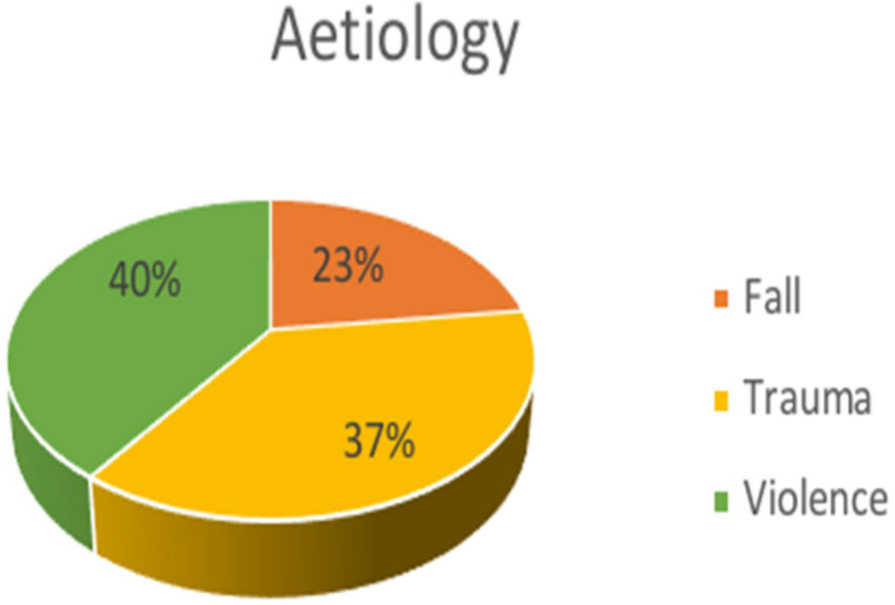
Distribution of the aetiology of fracture.

**Figure 3:**
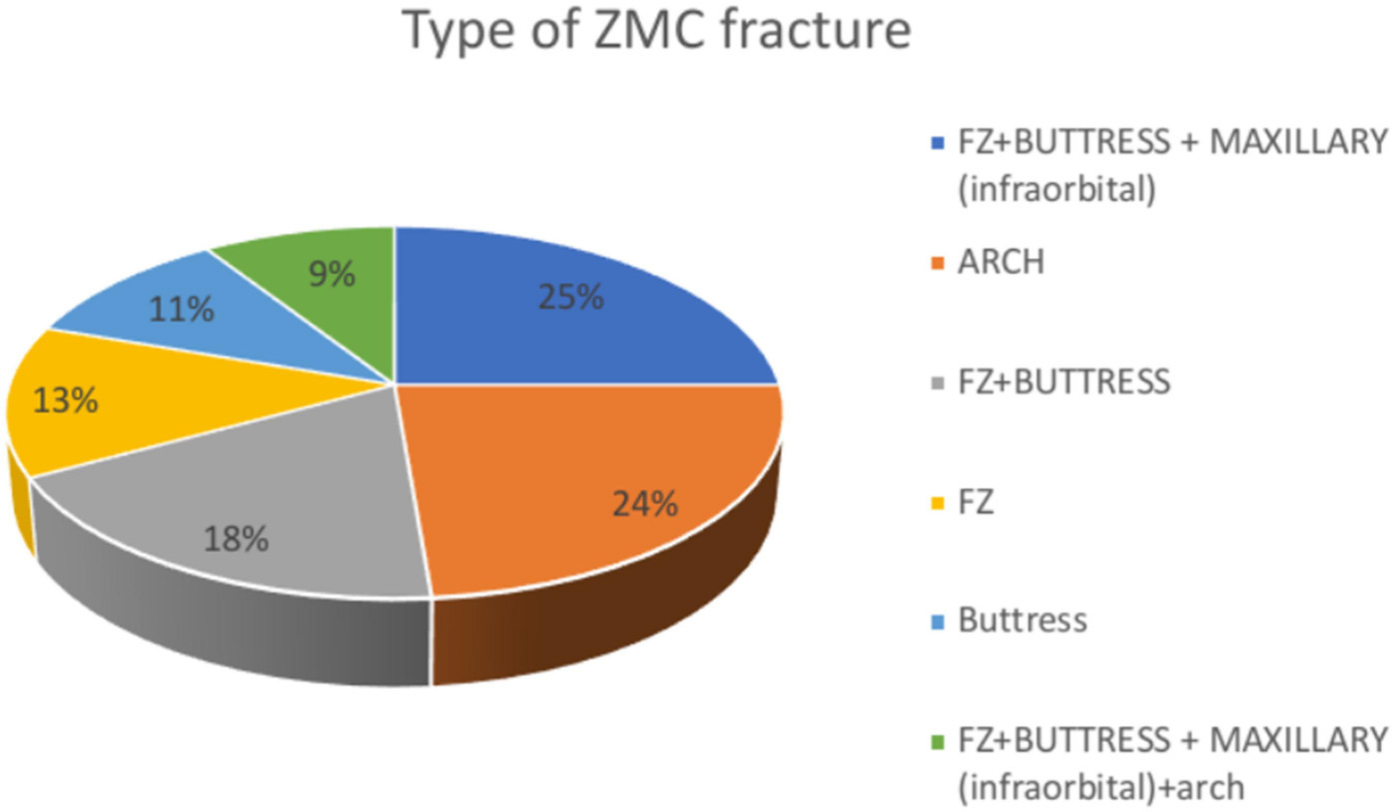
Distribution of ZMC fracture type.

A total of 100 patients with ZMC fractures were included in the present study. Out of these 100 patients, 34 subjects belonged to the 20–40 age group and the >60 age group, and 32 subjects belonged to the 41–60 age group. The mean age of the study subjects was 52.38 ± 18.07 years (Table 1). The study consisted of 50 males and 50 females, representing an equal proportion of male and female subjects (Figure 1). In the study group, Violence was the major reason reported (40 subjects), followed by trauma, in 37 subjects and fall, in 23 subjects (Figure 2). Frontozygomatic buttress fractures, combined with buttress fractures of the maxillary bone (infraorbital), were highly prevalent in 25% of the study sample, followed by zygomatic arch fractures (24%), and the least common was the FZ + Buttress + maxillary (infraorbital) + arch Type of fracture in 7% of the patients (Table 2). Conservative treatment was employed in 18% of the patients, while the remaining patients underwent surgery, with the most common methods being one-point fixation (18%) and three-point fixation (17%) (Table 3 and Figure 3). Table 4 compares the pre-operative and post-operative occurrence of paraesthesia by McNemar test. Pre-operatively, paraesthesia was reported by 18 subjects. Post-operatively, paraesthesia was reported by 18 subjects. There was a non-significant difference in the occurrence of pre-operative and post-operative paraesthesia. Table 5 compares the occurrence of post-operative paraesthesia among different age groups by Chi-square test. Post-operatively, there were six cases across each age group that reported paraesthesia. There was no significant difference in post-operative paraesthesia among different age groups. Table 6 compares the occurrence of post-operative paraesthesia among males and females. Post-operatively, paraesthesia was reported in 13 males and 5 females. Fischer extract test reveals no significant difference in post-operative paraesthesia among males and females. Table 7 compares the occurrence of post-operative paraesthesia according to the aetiology of fracture. Out of 23 patients whose fractures were due to a fall, 5 (21.7%) experienced paraesthesia, and 18 (78.3%) did not. Out of 37 patients whose fractures were due to trauma, 5 (13.5%) experienced paraesthesia, and 32 (86.5%) did not. Out of 40 patients whose fractures were due to violence, 8 (20%) experienced paraesthesia, and 32 (80%) did not. There was a non-significant association between fractures due to different causes and the development of post-operative paraesthesia as revealed by the Chi-square test.

**Table 1:** Distribution according to age groups.

Age group	*N*	Mean age
20–40	34	52.38 ± 18.07
41–60	32	
>60	34	

**Table 2:** Distribution of ZMC fracture type.

ZMC fracture type and treatment	*N*
FZ + BUTTRESS + MAXILLARY (infraorbital)	19
ZYGOMATIC ARCH	18
FZ (FRONTOZYGOMATIC) + MAXILLARY BUTTRESS	14
FZ (FRONTOZYGOMATIC)	10
MAXILLARY BUTRESS	8
FZ + MAXILLARY BUTTRESS + (infraorbital rim) + Zygomatic arch	7

**Table 3:** Distribution of treatment procedures done in ZMC fracture.

Treatment method	*N*
Conservative method	18
One point fixation	18
Three-point fixation	17
Two-point fixation	13
Three points	8
Three point/ arch (conservative)	8

**Table 4:** Comparison of pre-operative and post-operative paraesthesia.

Paraesthesia	Present	Absent	*p*-value
Pre-operative	18	82	1.000
Post-operative	18	82	

McNemar test; * for statistical significance; *p* value < 0.05.

**Table 5:** Comparison of post-operative paraesthesia among different age groups.

Age group	Present	Absent	*p*-value
20–40	6 (17.6%)	28 (82.4%)	0.991
41–60	6 (18.8%)	26 (81.3%)	
>60	6 (17.6%)	28 (82.4%)	

Chi-square test; * for statistical significance; *p* value < 0.05.

**Table 6:** Comparison of post-operative paraesthesia among males and females.

Gender	Present	Absent	*p*-value
Male	13 (26%)	37 (74%)	0.066
Female	5 (10%)	45 (90%)	

Fisher's exact test; * for statistical significance; *p* value < 0.05.

**Table 7:** Comparison of post-operative paraesthesia according to the aetiology of fracture.

Aetiology	Present	Absent	*p*-value
Fall	5 (21.7%)	18 (78.3%)	0.660
Trauma	5 (13.5%)	32 (86.5%)	
Violence	8 (20%)	32 (80%)	

Chi-square test; * for statistical significance; *p* value < 0.05.

## Discussion

The architectural frame of the zygomatic bone makes it possible to withstand the impacts of greater forces without giving way. When the impact is too high, it gets separated from the adjacent bones or nearby suture lines, leading to ZMC fractures. Depending on the velocity of impact they are seen as isolated or in association with other facial fractures because of the complex anatomy of the midface. The main goal is restoration of the preinjury configuration for treating ZMC fractures. Effective and successful repair needs accurate diagnosis and precise surgical exposure and reduction to fabricate the complex 3-dimensional anatomy. This article provides an overview of the epidemiology, aetiology, presentation, and management of surgically treated cases of ZMC fractures at our major trauma centre over a period of 3 years (12). Common causes include road accidents, assaults, falls, and sports injuries (13), with violence being the main cause at 40% in this study. Zygomatic arch fractures usually result from lateral impacts typical in assaults and sports, corroborating findings by Bogusiak, Arkuszewski (14), Ungari (15), and Zhang (13). Regionally, fracture causes vary; assaults account for 20%–64.5% globally (12–15), while traffic accidents (16–19) and sports injuries (15) also play roles. Literature shows ZMC fractures often involve multiple areas: the zygomaticomaxillary buttress, infraorbital rim, frontozygomatic suture, and zygomatic arch (16), matching our findings where 25% had multiple sites fractured and 24% had isolated arch fractures. The least common pattern involved four sites in 7%. Conversely, Ahmed (20) found the buttress fractured in 75% of cases, with other sites less frequent, likely due to trauma type—motor vehicle accidents vs. assaults in our study—shaping fracture patterns. Treatment-wise, 18% of patients had conservative management; most underwent surgery, with one-point fixation being most common (18%), followed by three-point fixation (17%). These align with Rohit et al. (16), who reported 16.3% non-surgical and 83.7% surgical treatments, with fixation points distributed as 22.9%, 42.4%, and 18.4%. Treatment complications are rare but include infraorbital nerve (ION) issues. Cakavicius (21) noted ION symptoms in 64.4%, depending on trauma severity. Foruzanfar (22) found the same rate of ION paresthesia, with 77% restoring full function post-treatment. Rohit et al. (16) observed persistent nerve symptoms in some cases. Our study also documented ongoing infraorbital nerve symptoms. Early intervention and proper fixation generally promote nerve recovery; persistent paresthesia may be due to misalignment or severe nerve damage. We found no significant differences in postoperative nerve symptoms across age or gender, consistent with Tabrizi et al. (23), who saw no gender-related differences in sensory outcomes at six months. The likelihood of postoperative paresthesia depends more on fracture location, especially involving the infraorbital canal or orbital floor, than on injury cause (24, 25). Most patients recover nerve function within three months after timely surgery and fixation (26). In our cases, stabilization was effective, with no bone displacement, plate loosening, or infections, indicating successful surgical and postoperative care.

The present study has few limitations. Retrospective study design, limited sample size for certain fracture subtypes, data collection from single center, and absence of long-term follow-up for both functional and esthetic outcomes. Reliance of medical records without standardized assessment of nerve injury may create potential bias.

## Conclusion

Zygomatic complex fractures are among the most frequently encountered injuries in maxillofacial trauma, with assaults being a leading cause. Despite advances in treatment, there remains ongoing debate about the best approaches for precise reduction, stabilisation, and fixation of these fractures. In conclusion, we found that violence was the predominant cause of ZMC fractures, affecting patients mainly in the 20–40 and over 60 age groups equally. For fractures without displacement, conservative management was effective. When surgery was required, fixation strategies varied using one-point, two-point, or three-point fixation depending on the severity and displacement of the fracture. Importantly, we observed that factors such as age, gender, or the cause of injury did not significantly affect the occurrence of paresthesia before or after treatment. Drawing from our clinical experience and study findings, it is clear that multiple fixation techniques can be successfully employed to stabilise ZMC fractures. The choice of method should be tailored to the specific fracture pattern and patient needs to ensure optimal outcomes.

## Limitations

1. Retrospective and single-centre design.
2. Limited sample size for certain fracture subtypes.
3. Absence of long-term functional/aesthetic follow-up.

## Data Availability

The original contributions presented in the study are included in the article/Supplementary Material, further inquiries can be directed to the corresponding author.
